# Differences in amino acid frequency in CagA and VacA sequences of *Helicobacter pylori* distinguish gastric cancer from gastric MALT lymphoma

**DOI:** 10.1186/s13099-016-0137-x

**Published:** 2016-11-08

**Authors:** Masahiko Hashinaga, Rumiko Suzuki, Junko Akada, Takashi Matsumoto, Yasutoshi Kido, Tadayoshi Okimoto, Masaaki Kodama, Kazunari Murakami, Yoshio Yamaoka

**Affiliations:** 1 Department of Environmental and Preventive Medicine, Faculty of Medicine, Oita University, 1-1 Idaigaoka, Hasama-machi, Yufu, Oita 879-5593 Japan; 2 Department of Gastroenterology, Faculty of Medicine, Oita University, 1-1 Idaigaoka, Hasama-machi, Yufu, Oita 879-5593 Japan; 3 Faculty of Welfare and Health Science, Oita University, 700 Dannoharu, Oita, Oita 870-1192 Japan; 4 Department of Medicine-Gastroenterology, Baylor College of Medicine and Michael E. DeBakey Veterans Affairs Medical Center, Houston, TX USA

**Keywords:** *Helicobacter pylori*, *cagA*, *vacA*, Amino acid polymorphism, Next generation sequencing

## Abstract

**Background:**

*Helicobacter pylori* is a pathogenic bacterium that causes various gastrointestinal diseases. The most common gastric malignancies associated with *H. pylori* are gastric cancer and lymphoma of mucosa associated lymphoid tissue (MALT). *Helicobacter pylori* virulence genes, namely *cagA* and *vacA,* are known to be associated with malignancy development. Conventionally, *cagA* and *vacA* were classified by looking at partial sequences of the genes. However, such genotyping has hardly proven useful predicting different risks for gastric cancer or MALT lymphoma. In search of new loci that distinguish these diseases, we investigated the full sequences of *cagA* and *vacA*.

**Results:**

We compared *cagA* and *vacA* sequences of 18 and 12 *H. pylori* strains obtained, respectively, from patients with gastric cancer and MALT lymphoma in Oita, Japan. Conventional genotyping of *cagA* and *vacA* showed no significant difference between the two diseases. We further investigated the full protein sequences of CagA and VacA to identify loci where allele frequency was significantly different between the diseases. We found four such loci on CagA, and three such loci on VacA. We also inspected the corresponding loci on the genes of 22 gastritis strains that potentially lead to gastric cancer or MALT lymphoma in the long run. Significant differences were observed at one CagA locus between gastritis and MALT lymphoma strains, and at one VacA locus between gastritis and gastric cancer strains.

**Conclusions:**

We found novel candidate loci in *H. pylori* virulence genes in association with two different types of gastric malignancies that could not be differentiated by conventional genotyping. Biological connotations of the amino acid polymorphisms merit further study.

**Electronic supplementary material:**

The online version of this article (doi:10.1186/s13099-016-0137-x) contains supplementary material, which is available to authorized users.

## Background

More than half of the world’s population is infected with *Helicobacter pylori*, a gram-negative spiral bacterium whose ecological niche is the human stomach. The infection is associated with severe gastritis-associated diseases, including peptic ulcer disease, gastric cancer (GC), and lymphoma of mucosa associated lymphoid tissue (MALT lymphoma) [1]. Most *H. pylori*-infected individuals develop histological gastritis, and about 10% of those infected develop severe diseases [2].

Epidemiological studies suggested that *H. pylori* infection plays an oncogenic role in both GC and MALT lymphoma [3–5]. Over their entire lifetimes, ~1 to 2% of *H. pylori* infected individuals develop GC, and less than 0.1% of them develop MALT lymphoma [6, 7]. The mechanisms for *H. pylori* involvement in these two kinds of malignant diseases remain unknown.

*Helicobacter pylori* is a highly heterogeneous species, and its virulence varies geographically [8]. The bacterial factors cytotoxin-associated gene A (CagA) and vacuolating cytotoxin A (VacA) are the most extensively studied *H. pylori* virulence factors. The major *H. pylori* virulence factor CagA is translocated into gastric cells via type IV secretion system and plays an important role in gastric carcinogenesis [9–11]. Injection of CagA requires a host cell receptor that was identified as integrin beta 1 [12, 13]. Recently there are several evidences that CagA also injected into B-cells in gastric MALT lymphoma [14, 15]. There are two types of clinical isolates: CagA-producing (*cagA*-positive) and CagA non-producing (*cagA*-negative) strains. The CagA protein contains a C-terminal region containing Glu-Pro-Ile-Tyr-Ala (EPIYA) tyrosine-phosphorylation motifs. The sequences are annotated according to segments (20–50 amino acids) flanking the EPIYA motifs (i.e., segments EPIYA-A, -B, -C, or -D) [16–18]. *cagA* containing EPIYA-D segments is typically observed in strains isolated from East Asia, and is denoted as East-Asian-type *cagA*; whereas *cagA* containing EPIYA-C segments is observed globally in non-East Asia strains, including those from Europe, South Asia, Africa, Australia, and the American continents, and is denoted Western-type *cagA* [19]. East-Asian-type *cagA* exhibits a stronger binding affinity for src homology-2 domain-containing phosphatase 2 (SHP2), and a greater ability to induce morphological changes in epithelial cells, than Western-type *cagA* [8, 16]. Our in vivo epidemiological studies also revealed a higher prevalence of East-Asian-type *cagA* strains in patients with GC than in patients with gastritis, and confirmed that East-Asian-type *cagA* is a significant risk factor for GC in Okinawa (Japan) and Thailand [20, 21]. Such different types of *H. pylori* virulence factor, at least in part, affect the geographic differences in incidence of gastric malignancies.

VacA is another extensively studied *H. pylori* virulence factor. Although nearly all strains possess the *vacA* gene. VacA protein posesses the capacity to induce cell vacuolization was found to differ significantly from strain to strain [22]. Four high sequence diversity regions of *vacA* have been found to closely associate with *H. pylori* vacuolating activity, including signal (s)-, intermediate (i)-, middle (m)-, and deletion (d)-regions. s, i, m, d regions are classified either type 1 or 2, respectively, and *vacA* s1m1 type is related with the higher risk of gastric malignancies [23, 24].

In Japan, almost all strains have ABD-type *cagA* and s1m1-type *vacA*, which are the most virulent genotypes, although clinical outcomes of the infection vary [25–27]. Conventional genotyping based on the partial sequences of the genes are not enough to predict different risks for gastric cancer or MALT lymphoma. Although the genetic diversity of these factors affects the pathogenesis of associated malignancies, few previous studies have focused on the entire sequences of *cagA* and *vacA* [28]. We therefore investigated the full protein sequences of CagA and VacA to identify amino acid loci where the allele frequency is significantly different between *H. pylori* strains isolated from patients with GC and MALT lymphoma. The differing amino acid residues were further compared with their corresponding amino acids in *H. pylori* strains isolated from patients with gastritis, who principally were individuals who may develop either GC or MALT lymphoma in the future.

## Methods

### Patients and *H. pylori*

*Helicobacter pylori* strains were isolated from the gastric mucosa of Japanese patients infected with *H. pylori* who underwent endoscopy at Oita University Faculty of Medicine Hospital (Yufu, Japan) and its affiliated hospitals (Oita, Japan) between February 1997 and April 2014. Included presentations were gastritis only (i.e., histological gastritis without peptic ulcers nor any malignancy), GC, and MALT lymphoma. We analyzed 18, 12, and 22 strains obtained from patients with GC, MALT, and gastritis, respectively. For patients with GC, MALT, and gastritis, respectively, the average age and the (male/female ratio) were 64.3 a (9/9), 64.4 a (8/4), and 57.9 a (9/13). Gastric biopsy specimens were taken from the antrum (pyloric gland area) and the corpus (fundic gland area). GC were identified by endoscopy; GC and MALT lymphoma were further confirmed by histopathology [29]. Written informed consent was obtained from all participants, and the protocol was approved by the Ethics Committees of Oita University Faculty of Medicine (Yufu, Japan).

### Isolation of *H. pylori*

Antral biopsy specimens were obtained for the isolation of *H. pylori* using standard culture methods, as previously described [25]. *Helicobacter pylori* DNA was extracted from confluent plate cultures expanded from a single colony using DNeasy Blood & Tissue kit (QIAGEN Inc., Valencia, CA, USA).

### Gene sequencing and data acquisition

Genome sequence data of *H. pylori* strains were obtained using paired-end reads (2 × 150 or 2 × 300 bp) on HiSeq 2000 and MiSeq sequencers (Illumina, Inc., San Diego, CA, USA). Sample multiplexing was provided by using an Indexed DNA library, and manipulated using the Nextera XT Index Kit and the Nextera XT DNA Library Preparation Kit (Illumina, Inc., San Diego, CA, USA), following the manufacturer’s instructions. We performed de-novo assembly of the short read data using the CLC Genomics Workbench v. 7.0.4 commercial software (CLC QIAGEN), with automated word size and bubble size, and a minimum contig size 200 bp. ORFs were predicted by the same software, which identified CagA and VacA using the genes of Japanese strain F32 (Accession Number NC_017366). We took top hit genes whose % identity and length coverage were at least 85 and 80%, respectively. In case *cagA* and *vacA* genes were not detected by next-generation sequencing, we used PCR and the Sanger method. Primers for PCR amplification and direct sequencing of the entire coding regions of *cagA* and *vacA* are shown in Table 1. The regions containing full-length *cagA* and *vacA* were amplified by PCR, and sequenced as previously described. We attached a list of accession numbers of the *cagA* and *vacA* sequences as Additional file 1.

**Table 1:** Primers used for DNA sequencing of *H. pylori cagA* and *vacA* in this study

Gene	Primer	Sequence (5′–3′)	References
*cagA*	Luni 1	ACATTTTGGCTAAATAAACACTG	[39]
	R5280	GTTGCACGCATTTTCCCTTGATC	[39]
	L2(+)	AAGGAGAAACAATGACTAACGAAACTATTG	[40]
	L2(−)	TCCTTTAAGATTTTTGGAAACCACCTTTTG	[40]
	hp552−R2	CTCTTACTCACCCTTGTATCCTCGCATA	In this study
	hp552−R1	GCCTGGATCGCTCAAACTTGGCATGC	In this study
	cagA−R2	GGGTGTTGATTTTAGACGGATC	In this study
	cagA−F9	TCGTTCAAGTTTTCCACCAAGTTGA	In this study
	cagA−F11	CAATCAAGAGGCTAGTAAGGAAG	In this study
	cagA−F6	CTCTCAAAGATTATGGGAAAAAA	In this study
	cagA−F10	ACAATAACGTTCTATCTTCTGTGCT	In this study
	cagA−F8	AAAGATCCGTCTAAAATCAACACCC	In this study
	TF	ACCCTAGTCGGTAATG	[20]
	TR	TATCAGAAGCTAAAAC	[20]
	OMF	AGCAAAAAGCGACCTTGAAA	[20]
	OMR	ATTCACGAGCTTGAGCCACT	[20]
*vacA*	vacAcysS−F2	GAATTTCAATGAAGAAGACTTGTT	In this study
	vacA−R4	GCGGTGTGTTTGTTGTATTTCCAT	In this study
	VA1−F	ATGGAAATACAACAAACACAC	[22]
	VAG−R	GCGTCAAAATAATTCCAAGGC	[25]
	VAG−F	CAATCTTGTCCAATCAAGCGAG	[25]
	VA3−F	GGTCAAAATGCGGTCATGG	[22]
	vacAfecE−R2	CTTATTGTTACTGGATGAGCCTA	In this study

### Comparison of amino acid frequency

We first investigated the genotypes of *cagA* and *vacA* by conventional motif analysis. East-Asian ABD-type *cagA* sequences (13 GC strains and 8 MALT strains), and s1m1-type *vacA* sequences (15 GC strains and 10 MALT strains) were deposited to GeneBank (Accession No. LC185348-LC185429) and used for further analysis. We aligned protein sequences of the genes using MAFFT v6.717b. We next investigated difference of amino acid allele frequency between GC and MALT strains for each locus using an original Perl script (detailed information available upon request). The significance of each amino acid difference was tested using Fisher’s exact test, as implemented by the R statistics software.

### MLST analysis

To investigate whether the observed differences in amino acid frequency could be explained by population structure, we used multi locus sequence typing (MLST) methods to construct a phylogenetic tree. Seven housekeeping genes of MLST (*atpA, efp, mutY, ppa, trpC, ureI,* and *yphC*) were obtained in the same manner as *vacA* and *cagA*. Specific portions of these genes were concatenated into sequences of 3406 bp each. We also downloaded from the PubMLST database 1439 reference strains with bacterial population information determined by STRUCTURE analysis in previous studies (http://pubmlst.org/helicobacter/). We next integrated the reference sequences with our sequences, and constructed a phylogenetic tree using MEGA v. 6.0.

## Results

### Conventional genotyping of *cagA* and *vacA*

We first analyzed the genotypes of these genes using DNA sequences obtained by next generation sequencing (NGS) or Sanger methods. The *cagA* genotype was classified by its combination of C-terminal EPIYA region segments denoted as A, B, C, and D. c*agA* with ABC segments was classified as Western type; *cagA* with ABD segments was classified as East-Asian type (Table 2). We analyzed *cagA* sequences of 14 GC and 11 MALT strains obtained by either NGS or the Sanger method. Most GC strains (13/14 strains; 92.9%) possessed East-Asian type ABD *cagA*, except for one *cagA*-negative strain. While most MALT lymphoma strains (8/11; 72.7%) also possessed East-Asian type ABD *cagA*, we also identified 1 Western type ABCC *cagA*, 1 AB type, and 1 atypical East-Asian ABABD type. Recent data revealed a strong non-random distribution of the B-motifs (including EPIYA and EPIYT) in CagAs. The EPIYT B-tyrosine phosphorylation motifs (TPMs) were significantly less associated with GC than the EPIYA B-TPMs [30]. In this study, most strains possessed EPIYA B-TPMs, except for three strains with ESIYA or ESIYT B-TPMs (one strain from GC had ESIYA B-TPM, and one strain from gastritis and one strain from MALT lymphoma had ESIYT B-TPM).

**Table 2:** Strain used for *cagA* analysis

Gene	Type	Gastric cancer (n = 14)	MALT lymphoma (n = 11)
*cagA*	ABD	13	8
	ABABD	0	1
	AB	0	1
	ABC	0	0
	ABCC	0	1
	Negative	1	0
Entire *cagA* studied	ABD	13	8

Sequences of *vacA* were categorized by their combinations of s, m, i, and d regions (Table 3). We analyzed *vacA* sequences of 17 GC and 12 MALT strains obtained by either NGS or the Sanger method. Each region was classified into two types, such as s1 and s2; each type 1 signified virulence, and each type 2 signified non-virulence. Most GC strains (15/17 strains: 88.2%) were s1m1i1d1-type, while most MALT lymphoma strains (10/12: 83.3%) were also s1m1i1d1-type. Somewhat interestingly, d2 genotypes were observed only in MALT lymphoma strains (2/12 strains). However, as expected, most strains isolated from Japanese patients with GC and MALT lymphoma were East-Asian-type *cagA* and s1m1i1d1 *vacA* genotypes; we could not find the genotypes specific for those diseases in Japan. We therefore investigated entire sequences of *cagA* and *vacA* genes to identify disease-specific loci at which significant differences in amino acid frequency were observed between GC and MALT lymphoma strains. Since different genotypes possessed different motifs and repeats, we focused on *cagA* with ABD segments and *vacA* of s1m1i1d1 type, which possessed stronger virulence than other genotypes, and predominate among East Asian *H. pylori*.

**Table 3:** Strain used for *vacA* analysis

Gene	Type	Gastric cancer (n = 17)	MALT lymphoma (n = 12)
*vacA*	s1m1i1d1	15	10
	s1m2i1d1	2	0
	s1m2i1d2	0	1
	s1m2i2d2	0	1
Entire *vacA* studied	s1m1i1d1	15	10

### Significant difference of amino acid frequency between samples of GC and MALT lymphoma

**Fig. 1:**
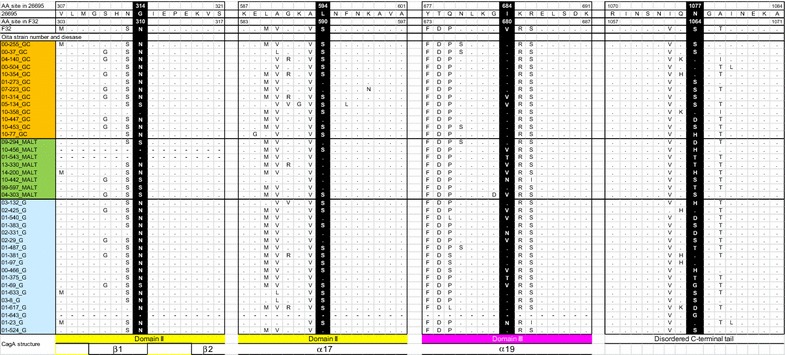
CagA amino acid comparison between MALT lymphoma, gastric cancer, and gastritis strains. CagA amino acid comparison of strains 26,695, F32, and clinical isolates obtained in Oita, Japan are presented. Four significantly different loci are shown in *white letters* with *black background*. CagA structure was referred to [31]. *GC* gastric cancer, *G* gastritis

We detected disease-specific differences at four *cagA* loci in strain 26,695 (sites 314, 594, 684, and 1077) (left half of columns in Table 4; Fig. 1). At *cagA* site 314, asparagine (N) was dominant in the GC group, while serine (S) was the majority state in the MALT group. At site 594 of *cagA*, serine (S) was dominant in the GC group, while leucine (L) was the majority state in the MALT group. At site 684 of *cagA*, isoleucine (I) was dominant in the GC group, whereas the MALT group exhibited four amino acid variants, with valine (V) the most common among them. Among six kinds of amino acid substitution at *cagA* site 1077, serine (S) was dominant in the GC group; residues other than asparagine (N) exhibited similar frequencies in the MALT group. All four of these CagA loci differed significantly (P < 0.05, Fisher’s exact test) between the 13 GC samples and the 8 MALT lymphoma samples.

**Table 4:** Difference of amino acid frequency at loci: comparison of each disease in genotypes of *cagA* ABD type

Position		Amino acid residue	Gastric cancer (GC) (n = 13)	MALT (n = 8)	P value	Gastritis (G) (n = 18)	P value	
26,695	F32				GC vs MALT		G vs GC	G vs MALT
314G	310N	G	1	0	0.017	0	0.71	0.0078 (P < 0.01)
		N	11	2		16		
		S	1	4		1		
		Sequence gap	0	2		1		
594L	590S	L	3	6	0.032	5	1	0.081
		S	10	2		12		
		Sequence gap	0	0		1		
684I	680V	I	11	2	0.011	9	0.33	0.63
		N	0	1		2		
		T	0	1		1		
		V	2	4		5		
		Sequence gap	0	0		1		
1077N	1064S	D	1	1	0.029	3	0.58	0.46
		H	1	2		2		
		N	4	0		4		
		S	7	2		5		
		T	0	3		2		
		G	0	0		2

**Fig. 2:**
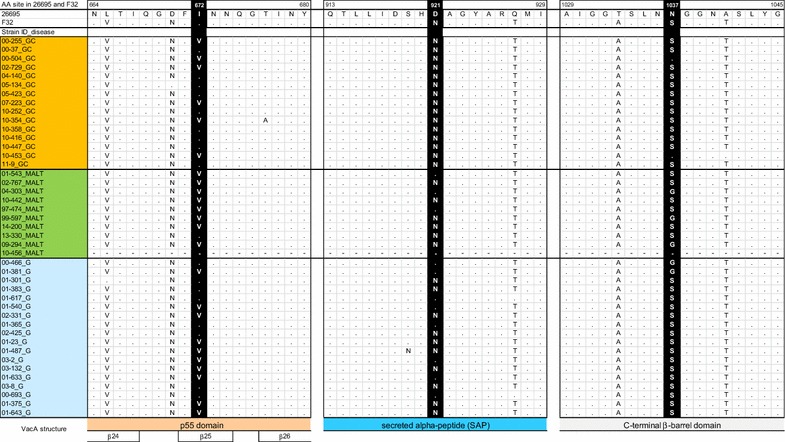
VacA amino acid comparison between MALT lymphoma, gastric cancer, and gastritis strains. VacA amino acid comparison of strains 26,695, F32, and clinical isolates obtained in Oita, Japan are presented. Three significantly different loci are shown in *white letters* with *black background*. VacA structure was referred to [32]. *GC* gastric cancer, *G* gastritis

We detected significant differences in three *vacA* loci at sites 672, 921, and 1037 in strain 26,695 (left half of Table 5; Fig. 2). At *vacA* site 672, isoleucine (I) was dominant in the GC group, while valine (V) was dominant in the MALT group. At *vacA* site 921, asparagine (N) predominated in both the GC and the MALT groups; however, some aspartic acid (D) substitutions occurred in the MALT group. At position 1037 of *vacA*, serine (S) predominated in both groups, but the frequencies of asparagine (N) and glycine (G) differed. All three of these VacA loci differed significantly (P < 0.05, Fisher’s exact test) between the 15 GC samples and the 9 MALT lymphoma strains.

**Table 5:** Difference of amino acid frequency at loci: comparison of each disease in genotypes of *vacA* s1m1 type

Position		Amino acid residue	Gastric cancer (GC) (n = 15)	MALT (n = 10)	P value	Gastritis (G) (n = 18)	P value	
26,695	F32				GC vs MALT		G vs GC	G vs MALT
672I	672I	I	9	1	0.033	8	0.49	0.19
		V	6	8		10		
		Sequence gap	0	1		0		
921D	921N	D	0	3	0.042	8	0.0036 (P < 0.01)	0.69
		N	15	6		10		
		Sequence gap	0	1		0		
1037N	1037S	S	13	6	0.042	16	0.16	0.30
		N	2	0		0		
		G	0	3		2		
		Sequence gap	0	1		0

### Absence of population structure confirmed by MLST phylogeny

If the samples were taken from different populations and disease prevalence was different between the populations, amino acid frequency could differ not because of the diseases but because of the populations. To test for structure within the studied strain population, we constructed phylogenetic trees based on MLST sequences. MLST uses seven housekeeping genes; therefore, a phylogenetic tree based on MLST reflects lineage differences that are independent of virulence. If adenocarcinoma strains and lymphoma strains were divided into two clusters in the MLST tree, amino acid differences may be irrelevant to disease type.

In the MLST phylogenetic tree with global strains, both GC and MALT lymphoma groups were located around East Asian strains (Additional file 2: Figure S1). The phylogenetic tree of only Japanese strains exhibited a radial branching shape, and GC and MALT lymphoma strains scattered randomly (Additional file 3: Figure S2). These trees imply an absence of population structure among the GC and MALT lymphoma samples.

### Significant differences in loci between GC or MALT lymphoma and gastritis strains

The significant disease-specific *cagA* and *vacA* variations were not due to sample population differences. We therefore compared loci in gastritis samples with their counterparts in GC and MALT lymphoma samples; these samples were obtained at the same hospitals in Japan. Both of the malignant diseases were presumed to be derived from gastritis, so we examined the same *cagA* and *vacA* loci for 22 gastritis strains, and compared these observations with corresponding amino acid frequencies for GC and MALT lymphoma samples.

Four *cagA* loci in gastritis samples (positions 314, 594, 684, and 1077) were checked (right half of Table 4; Fig. 1). We analyzed *cagA* sequences of 18 gastritis strains obtained by either NGS or the Sanger method. Two *cagA* positions (684 and 1077) in the gastritis samples were not significantly different from their counterparts in GC or MALT lymphoma samples. Amino acid frequency of gastritis samples seems to be intermediate between GC and MALT lymphoma samples. However, *cagA* position 314 in gastritis samples was significantly different (P = 0.008, Fisher’s exact test) from its counterpart in MALT lymphoma samples, but very similar to GC samples (P = 0.71). Also, *cagA* 594 in gastritis samples had the same tendency of near significant difference from MALT lymphoma samples (P = 0.081), but was indistinguishable from GC samples (P = 1.0). From this analysis, *H. pylori cagA* in patients with MALT lymphoma had the residues 314S and 594L, whereas the patients with gastritis and GC patient often had the residues 314N and 594S.

We next checked three *vacA* loci (positions 672, 921, and 1037) in gastritis samples (right half of Table 5; Fig. 2). Since we could not obtain *vacA* sequences from four gastritis strains (these strains lacked *cagA*), 18 sequences were analyzed. Two *vacA* positions (672 and 1037) of gastritis samples were not significantly different from their GC or MALT lymphoma counterparts. However, one *vacA* position (921) differed significantly between gastritis samples and GC samples (P = 0.004, Fisher’s exact test). This position did not differ significantly between gastritis samples and MALT lymphoma samples (P = 0.69). The *H. pylori vacA* from patients with gastritis and MALT lymphoma assumed either a D or N state at position 921; the homologous position in GC patient *H. pylori* was a conserved N.

## Discussion

We found disease-specific amino acid variations at four loci in ABD-type CagA (positions 314, 594, 684, and 1077 in strain 26,695), and at three loci in s1m1-type VacA (positions 672, 921, and 1037), some of which deviated significantly between GC strains and MALT lymphoma strains. There were no differences in the amino acid frequency in all four CagA loci between GC and gastritis. Since gastritis cases in this study comprised atrophic gastritis—the precancerous phase—these results were not so surprising. However, CagA position 314 was significantly different between MALT lymphoma and gastritis strains. Most GC and gastritis strains contained asparagine (N) at position 314, but MALT lymphoma strains contained mainly serine (S) (4/6 sequenced strains). This difference suggested that N to S substitution at site 314 might be involved with the development of MALT lymphoma.

Recent studies have suggested that CagA would play a pivotal role in gastric MALT lymphoma pathogenesis [9, 14, 15]. CagA was indicated to translocate into human B-lymphocytes, hindering their apoptosis. This CagA translocation presumably leads to continuous B-lymphoid cell proliferation. Genetic variations of CagA in the development of MALT lymphoma are still unknown, although diversity has been indicated to affect the extent to which CagA causes inflammation to gastric epithelial cells. Here, we report disease-specific CagA sites that may discriminate between GC and MALT lymphoma.

**Fig. 3:**
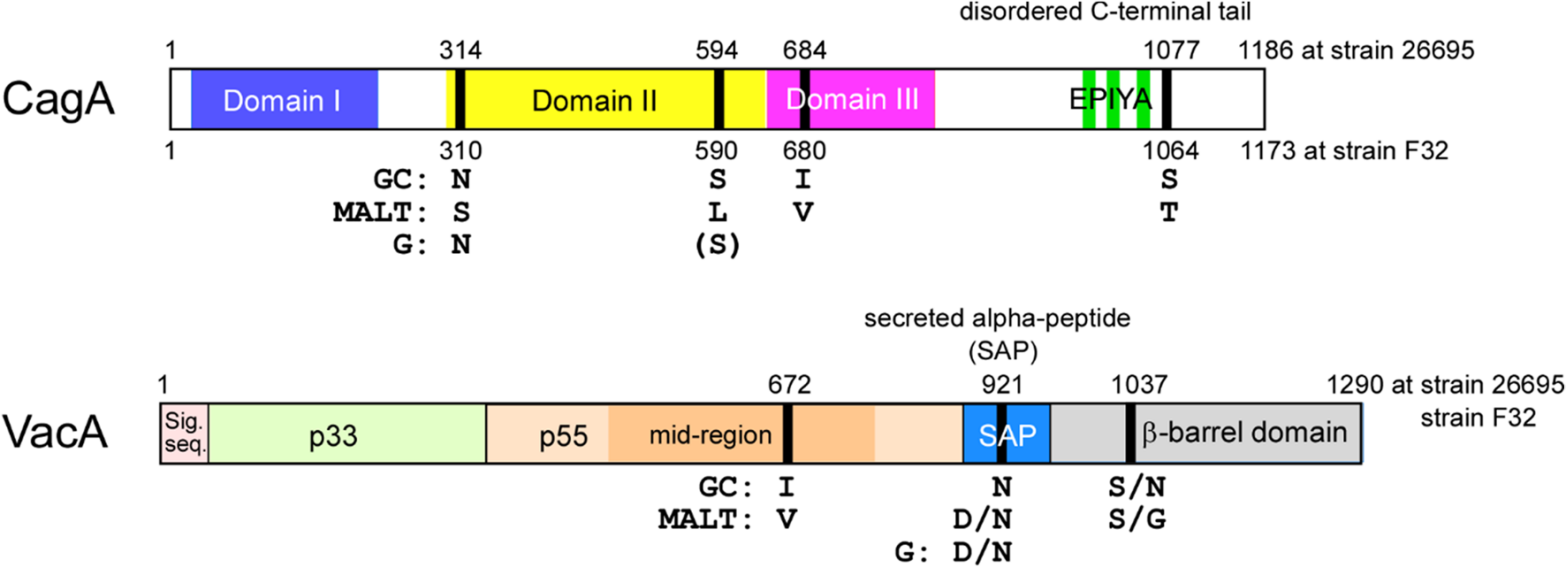
Structures of CagA and VacA. Amino acid variations at four loci in ABD-type CagA (positions 314, 594, 684, and 1077 in strain 26,695) and those at three loci in s1m1-type VacA (positions 672, 921, and 1037); significant differences between gastric cancer strains and MALT lymphoma strains are presented

**Fig. 4:**
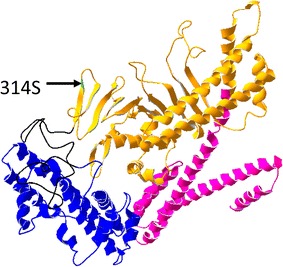
Structural homology modeling of CagA. Three-dimensional structure of CagA derived from 09-294 (MALT lymphoma) was deduced by SWISS-MODEL [37, 38]. This model was constructed from the amino acid sequence of 09-294 from (residues 26F–819S), using 4dvy.1.A as a template. Domain I (26F–219F) is drawn in blue, Domain II (310G–638E) in yellow, and Domain III (644K–819S) in magenta. The arrow indicates the significant amino acid substitution 314S in MALT lymphoma, which is located in Domain II

From the view point of the CagA tertiary structure, deduced from the protein structure of the 26,695 reference strain was reported [31], and residue 314 is located in the middle region of CagA, in Domain II of β-sheet 1, continuing from N-terminal Domain I; position 594 is located in α-helix 17 of Domain II (Figs. 1, 3). Both loci are close to the basic amino acid cluster at α-helix 18 in Domain II where mediates CagA membrane association, so near plasma membrane of host cells. We also used structural homology modeling of MALT lymphoma strain (09-294) CagA (Fig. 4) to place the 314S residue in a solvent-exposed region of CagA. A disease-specific polymorphism at this site may easily interact with other downstream signal molecules just under the plasma membrane in B-lymphocytes. Tyrosine phosphorylation of EPIYA/T B-TPMs were reported to be involved in the regulation of host cell signaling cascades and interacted with epithelial cells [30]. In this study, most strains possessed EPIYA, not EPIYT B-TPMs, which were in agreement with the previous report that most East Asian strains (91.1%) possessed EPIYA B-TPMs [30].

One of the three significant VacA loci, position 672 is located in p55 domain. VacA p55 plays an important role in mediating VacA binding to host cells and the crystal structure of VacA p55 domain had already been reported using strain 60,190 [32]. The position 672 (corresponding to position 636 in strain 60,190) is located in middle region at the 25th β-helix structure from N-terimus of VacA p55 (Table 2), exposing to outside of p55 molecule in the 3D-model of VacA p55 of strain 60,190. It is unclear whether the function of VacA can be different between Valine (mainly in MALT lymphoma) and Isoleucine (mainly in GC) in this position. However, the most divergent amino acids among VacA molecule were located in surface-exposed residues within the p55 domain; therefore these residues should be involved in the optimized binding of VacA to different receptors or targets in host cells [33]. Several VacA receptors in host cells have been unveiled recently [34], and we hope that the binding position of p55 domain to each receptor will be clarified in the future studies.

The second VacA loci obtained from comparing GC and MALT lymphoma sequences, position 921 was interestingly, also highly significant between GC and gastritis. The amino acid at this position in all 15 GC strains was asparagine (N). Among gastritis and MALT strains, this site alternated between D and N (D: 8 gastritis, 3 MALT strains; N: 10 gastritis, 6 MALT strains). Reference Japanese strain F32, which derived from a GC case, also had 921N. These observations suggested that D to N at position 921 at later stages of gastritis might be involved with the development of GC. Position 921 is located within the secreted alpha-peptide (SAP) region (Figs. 2, 3). SAP is cut and secreted from *H. pylori* with the mature VacA p88 [32, 35]. Hydrophobicity of asparagine (N) may possibly affect differently from hydrophilic aspartic acid (D) in this small peptide of unknown function. Detailed studies including on function of SAP are necessary to understand this 921N preference in VacA of GC strains.

This study had several limitations. First, the sample number was small. Although we found several statistically different loci between GC and MALT lymphoma, the differences were marginal, and their biological importance was unclear. In this study, we focused on the two major virulence factors; however, *H. pylori* genomes contain more than 1500 genes. If we were to compile the amino acid differences across entire *H. pylori* genomes between different diseases, some of the resulting loci may be polymorphisms important to the development of specific diseases. Further studies using isogenic mutants of the loci may be necessary to clarify the function of these amino acid differences. Second, many gastritis cases contained gastric atrophy since most infected Japanese had gastric atrophy in some degree. Therefore, most loci seemed to show no difference in amino acid frequency between GC and MALT lymphoma sequences. Although NGS technology is proposed wide variety of appreciation for biological problems [36], there are also some limitations to using currently available NGS data. In most cases, NGS data alone could not obtain even the full *cagA* and *vacA* sequences; classical Sanger methods were necessary to confirm these sequences. The accuracy of NGS data is improving; in the near future we will use NGS to search for novel polymorphisms related to specific gastroduodenal diseases.

Despite these limitations, our methodology does offer a promising means of finding novel *H. pylori*-polymorphisms associated with specific diseases that involves NGS analysis.

## Additional files

**Additional file 1: Table S1.** Accession numbers of the *cagA* and *vacA* gene sequences.

**Additional file 2: Figure S1.** Phylogenetic tree of the 18 GC and 12 MALT strains with 1439 PubMLST strains. This tree shows 18 GC and 12 MALT strains in 1439 global strains obtained from the PubMLST database. Orange and green triangles represent GC and MALT strains, respectively. hspEAsia, hspMaori, and hspAmerind are subpopulations of hpEastAsia. All GC and MALT strains were included within hpEastAsia population. (NJ-tree, Kimura-2 parameters, MEGA v. 6.0.).

**Additional file 3: Figure S2.** Phylogenetic tree of only 18 GC and 12 MALT strains. The star-like topology of this tree implies that these strains are genetically homogenous, and that their population has no clear structure (NJ-tree; Kimura-2 parameters; MEGA v. 6.0).

## Abbreviations

MALT: mucosa associated lymphoid tissue
GC: gastric cancer
CagA: cytotoxin-associated gene A
SHP2: src homology-2 domain-containing phosphatase 2
MLST: multi locus sequence typing
NGS: next generation sequencing
