# Supplementary material for: Differences in amino acid frequency in CagA and VacA sequences of Helicobacter pylori distinguish gastric cancer from gastric MALT lymphoma
Source: Gut Pathog. 2016 Nov 8;8:54. doi: 10.1186/s13099-016-0137-x (PMC5101760; doi:10.1186/s13099-016-0137-x)
Supplement: Supplementary file 2 — Additional file 2: Figure S1. Phylogenetic tree of the 18 GC and 12 MALT strains with 1439 PubMLST strains. This tree shows 18 GC and 12 MALT strains in 1439 global strains obtained from the PubMLST database. Orange and green triangles represent GC and MALT strains, respectively. hspEAsia, hspMaori, and hspAmerind are subpopulations of hpEastAsia. All GC and MALT strains were included within hpEastAsia population. (NJ-tree, Kimura-2 parameters, MEGA v. 6.0.). [file 13099_2016_137_MOESM2_ESM.pptx]

## Slide 1
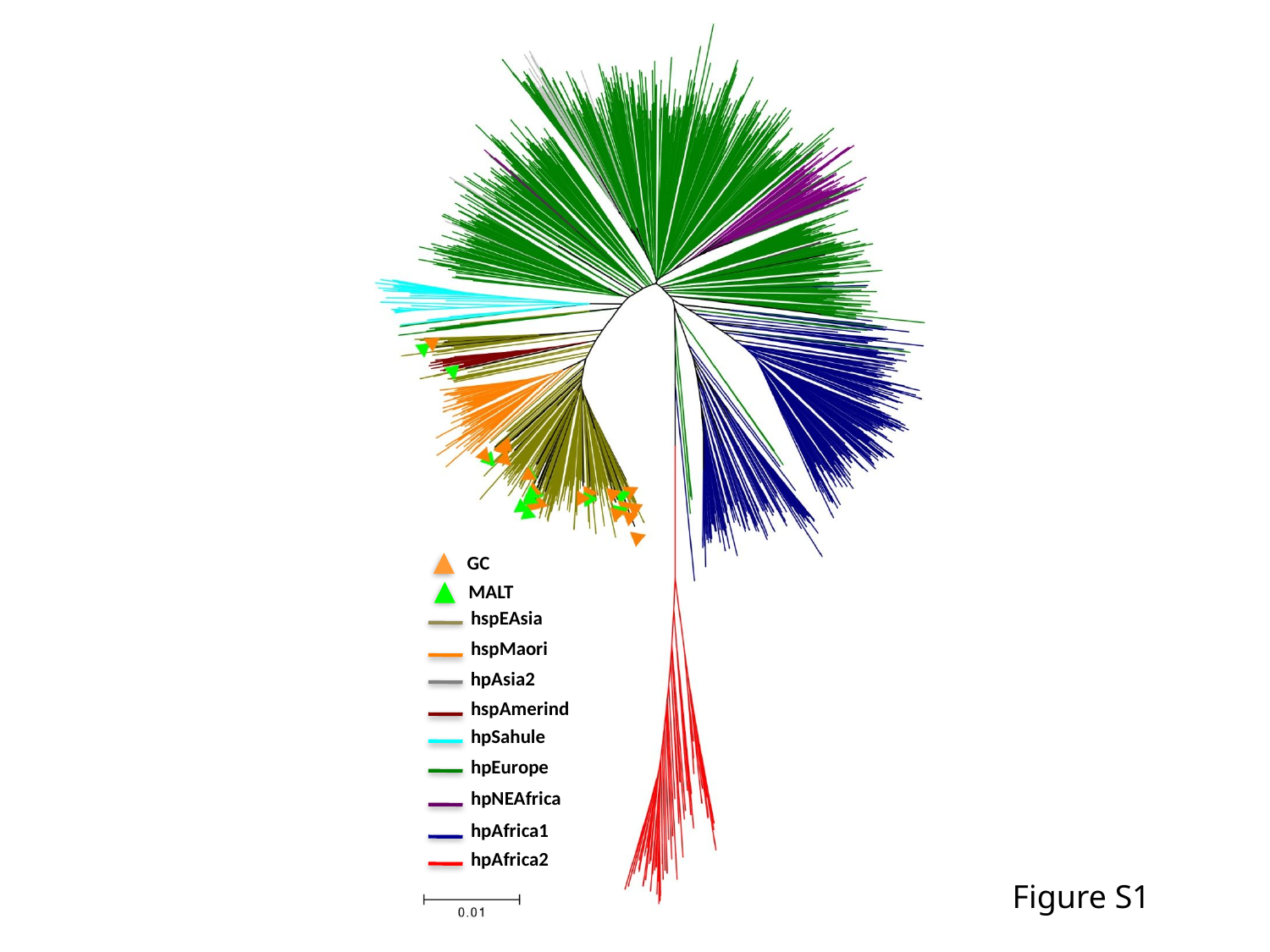

GC
MALT
hspEAsia
hspMaori
hpAsia2
hspAmerind
hpSahule
hpEurope
hpNEAfrica
hpAfrica1
hpAfrica2
Figure S1
