# Supplementary figures and images for: Differences in amino acid frequency in CagA and VacA sequences of Helicobacter pylori distinguish gastric cancer from gastric MALT lymphoma
Source: Gut Pathog. 2016 Nov 8;8:54. doi: 10.1186/s13099-016-0137-x (PMC5101760; doi:10.1186/s13099-016-0137-x)

## Slide 1
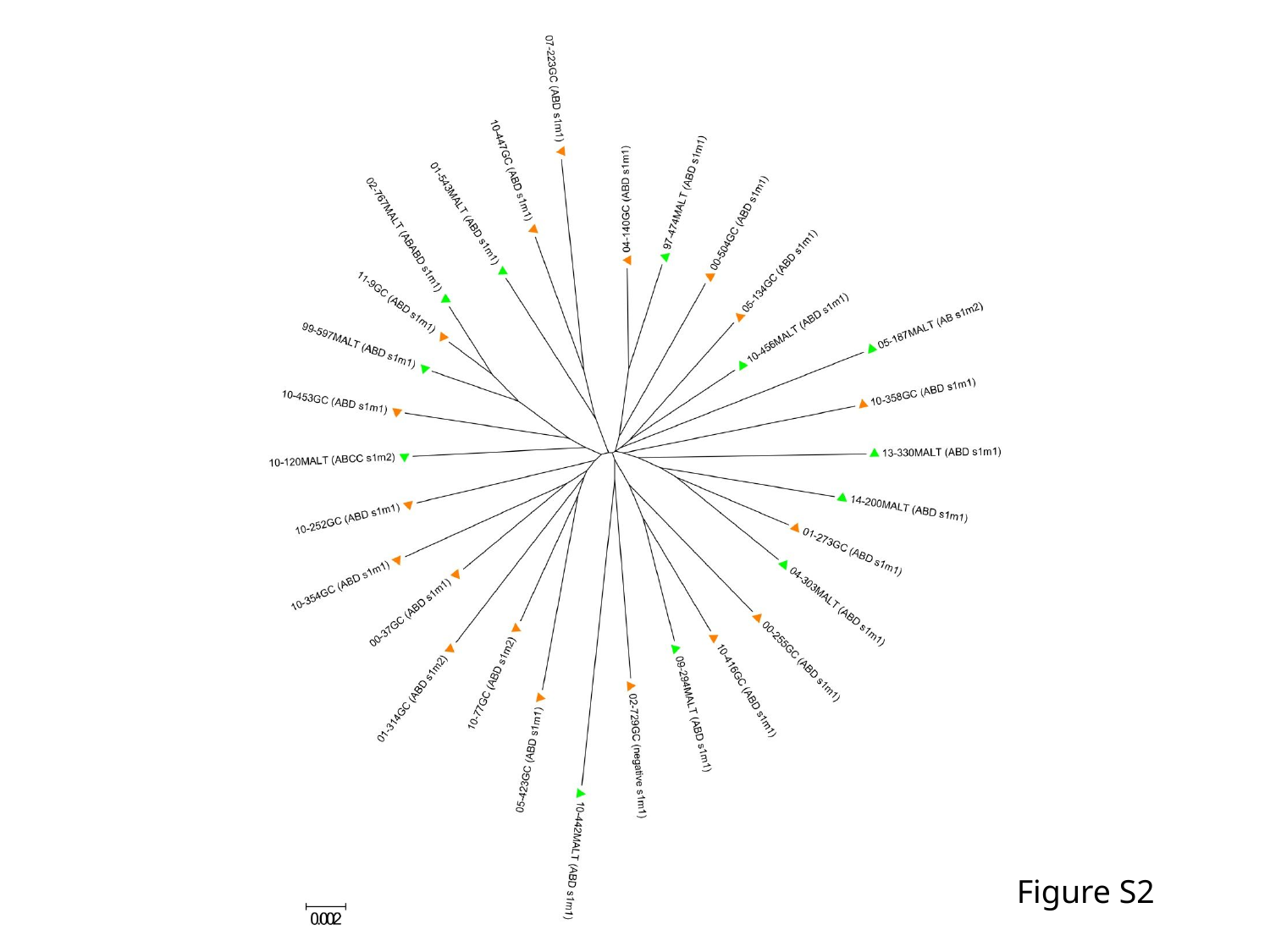

Figure S2

Supplement: Supplementary file 3 — Additional file 3: Figure S2. Phylogenetic tree of only 18 GC and 12 MALT strains. The star-like topology of this tree implies that these strains are genetically homogenous, and that their population has no clear structure (NJ-tree; Kimura-2 parameters; MEGA v. 6.0). [file 13099_2016_137_MOESM3_ESM.pptx]
